# Skin Transcriptome Profiles Associated with Skin Color in Chickens

**DOI:** 10.1371/journal.pone.0127301

**Published:** 2015-06-01

**Authors:** Jianqin Zhang, Fuzhu Liu, Junting Cao, Xiaolin Liu

**Affiliations:** 1 College of Animal Science and Technology, Northwest A&F University, Yangling, Shaanxi, China; 2 Shaanxi Key Laboratory of Molecular Biology for Agriculture, Yanging, Shaanxi, China; University of Lausanne, SWITZERLAND

## Abstract

Nutritional and medicinal benefits have been attributed to the consumption of tissues from the black-boned chickens in oriental countries. Lueyang black-boned chicken is one of the native chicken breeds. However, some birds may instead have white or lighter skin, which directly causes economic losses every year. Previous studies of pigmentation have focused on a number of genes that may play important roles in coat color regulation. Illumina2000 sequencing technology was used to catalog the global gene expression profiles in the skin of the Lueyang chicken with white versus black skin. A total of 18,608 unigenes were assembled from the reads obtained from the skin of the white and black chickens. A total of 649 known genes were differentially expressed in the black versus white chickens, with 314 genes that were up regulated and 335 genes that were down-regulated, and a total of 162 novel genes were differentially expressed in the black versus white chickens, consisting of 73 genes that were up-regulated (including 4 highly expressed genes that were expressed exclusively in the skin of the black chickens) and 89 genes that were down-regulated. There were also a total of 8 known coat-color genes expressed in previous studies (*ASIP*, *TYR*, *KIT*, *TYRP1*, *OCA2*, *KITLG*, *MITF* and *MC1R*). In this study, 4 of which showed greater expression in the black chickens, and several were up-regulated, such as *KIT*, *ASIP*, *TYR* and *OCA2*. To our surprise, *KITLG*, *MITF* and *MC1R* showed no significant difference in expression between the black- and white-skinned chickens, and the expression of *TYRP1* was not detected in either skin color. The expression of *ASIP*, *TYR*, *KIT*, *TYRP1*, *OCA2*, *KITLG*, *MITF* and *MC1R* was validated by real-time quantitative polymerase chain reaction (qPCR), and the results of the qPCR were consistent with the RNA-seq. This study provides several candidate genes that may be associated with the development of black versus white skin. More importantly, the fact that the *MC1R* gene showed no significant difference in expression between the black and white chickens is of particular interest for future studies that aim to elucidate its functional role in the regulation of skin color.

## Introduction

The skin color of chickens is an important economic trait. Normally, there are three skin colors found in chickens: white, yellow and black. Skin color is the most direct marker whether the bird is black-bone chicken or not. In oriental countries, nutritional and medicinal benefits have been attributed to the consumption of tissues from the black-boned chickens for thousands of years. Therefore, skin color is a key trait that contributes to significant economic value in terms of poultry production. The Lueyang black-boned chicken is one of the native chicken breeds of Lueyang County in the Shaanxi Province of China. This bird is typically composed of eight characteristic black parts: feathers, wing tips, beak, cockscomb, skin, bones, legs and claws. However, some birds may instead have white or lighter skin, which directly affects the selective breeding of the Lueyang chicken population and causes economic losses every year. The presence of pigmented skin among Lueyang chickens is significantly tied to their economic value and the speed of breeding.

Pigmentation is a complex trait that depends on genetics and other factors, including the environment and certain drugs [1–3]. In vertebrates, melanic coloration is often genetically determined and associated with various behavioral and physiological traits, suggesting that some genes involved in melanism may have pleiotropic effects [4]. Many genes have been found to play well-known roles in pigmentation, based on previous genome-wide association scans (GWAS), and the analysis of these genes has identified many single nucleotide polymorphisms (SNPs), e.g., *ASIP*, *OCA2*, *TYR*, *MC1R*, *KITLG*, *TYRP1*, *SLC24A4*, *MITF* and *KIT* [5–9]. Several previous studies have paid significant attention to the coat color of animals and showed that this color is determined by the amount and type of melanin produced and released by the melanocytes present in the skin. For example, recent studies revealed that *MC1R* and *ASIP* are major genes involved in determining coat color in sheep [10,11], and *TYRP1* is a strong candidate gene for color variation in Soay sheep [12]. An expression analysis was performed on 10 genes related to melanocyte development in Silky Fowl and White Leghorn embryos, via qRT-PCR, and a regulatory network for melanocyte development was constructed based on the expression data [13]. Emaresi et al. (2013) considered that color variation was likely to stem from differences in the expression levels of genes belonging to the melanocortin in the tawny owl [14]. Previous studies only focused on the gene expression patterns in animals with different coat color. However, in our study, even the chick had the same coat color, but the skin color was different. The aim of this work was to study the transcriptome profiles in the skin of chickens with black versus white skin using high-throughput RNA deep-sequencing technology, to investigate the different expression profiles of the genes involved in skin pigmentation, then look for the main differences between black and white skin colors in Lueyang chickens. This will enable us to understand the molecular mechanisms involved in skin pigmentation and provide a valuable theoretical basis for the selection of the black trait during the selective breeding of the Lueyang chick.

## Materials and Methods

### Ethics Statement

All of the animals were handled in strict accordance with good animal practices as defined by the relevant national and/or local animal welfare bodies. The experimental procedure was approved by the Animal Care and Use Committee of Northwest A&F University, China and was performed in accordance with the animal welfare and ethics guidelines.

### Experimental Design and Chick Skin Sampling

A total of 200 eggs were collected at random from the Lueyang chicken core breeding factory, Lueyang County, Shaanxi Province, China. The incubation and care of the birds were completed at the poultry farm at Northwest Agricultural and Forestry University, Shaanxi, China. Ten healthy, 16-week-old white and black female Lueyang chickens (5 birds per color) were selected for the sample collection. A piece of skin (8 mm in diameter) from the left back was collected and immediately placed in liquid nitrogen. Total RNA was extracted from the sample using Trizol reagent (Takara, Dalian, China) according to the manufacturer’s instructions. The RNA integrity was evaluated using gel electrophoresis, and the RNA purity was checked via the OD 260/280 ratio and the RIN value. RNA samples with a RIN value greater than 8.0 and an OD 260/280 ratio greater than 1.9 were selected for deep sequencing. According the result of sequencing, some colored gene expressions were validated using Real time quantitative polymerase chain reaction (qPCR). *β-actin* was used as housekeeping gene.

### Library Generation and Sequencing

Three RNA samples from either the black or white skin samples were pooled following mRNA isolation. The isolated mRNA was fragmented, followed by first-strand cDNA synthesis using random hexamer primers. The second-strand cDNA was synthesized using buffer (Invitrogen, 20 μL), dNTPs (0.25 mM / μL), RNaseH (0.05 U / μL) and DNA polymerase I (0.5 U / μL). The short cDNA fragments were purified using the QIAQuick PCR extraction kit (LianChuan Sciences, Hangzhou, China). The fragment ends were repaired, A-tailed and ligated to sequencing adaptors. Suitably sized (350 ± 50 bp) fragments were selected following an agarose gel electrophoresis and used as templates for the PCR amplification to generate an RNA-seq library. The sequencing of the library was performed using an Illumina HiSeq 2000 (LianChuan Sciences, Hangzhou, China). The raw reads were cleaned by removing the adaptors and low quality reads prior to assembly. The unigene assembly was carried out using the short reads assembly program. All assembled unigenes were compared with the proteins in the non-redundant (nr) protein database, using BLAST software with a significance threshold of E-value < 10^-5^. Functional categorization by gene ontology (GO) terms was carried out according to molecular function, biological process and cellular component ontologies with an E-value threshold of 10^-5^. The pathway assignments were performed by sequence searches against the Kyoto Encyclopedia of Genes and Genomes (KEGG) database and using the BLASTX algorithm with an E-value threshold of 10^-5^.

### Differential gene expression profiling

The expression abundance of each assembled transcript was measured through Fragments per Kilobase of exon model per Million mapped reads (FPKM) values. All reads were mapped onto the non-redundant set of transcripts to quantify the abundance of assembled transcripts. Bowtie was used for read mapping and applied for FPKM based expression measurement. The expressions of each reads between sample pairs (BS vs WS) were calculated using the numbers of reads with a specific match. Between the two samples, a minimum of a two-fold difference in log 2 expression were considered as expression differences.

### Real-time quantitative RT—PCR

The expression of these genes was quantified by qRT-PCR using QuantiTect SYBR Green RT-PCR (Qiagen, Waltham, MA). Information regarding the primers of *MC1R*, *TYR*, *KIT*, *ASIP*, *TYRP1*, *OCA2*, *KITLG*, *MITF* used for the qPCR can be found in Table 1. β-actin was used as housekeeping gene. Quantitative real-time PCR was performed in triplicate on the Stratagene iQ5 system. The 12.5 μL PCR reaction included 6.25 μL SYBR Premix Ex Taq II (TaKaRa, Dalian,China), 0.25 μL (10 p moL / μL) specific forward primer, 0.25 μL (10 p moL / μL) reverse primer, 0.5 μL ROX reference dye, 0.25 μL (10 ng / μL) diluted cDNA and 5.25 μL RNase free water. Cycling parameters were 95°C for 10 min, followed by 37 cycles of 95°C for 15 sec, 57°C for 30 sec and 72°C for 45 sec. Melting curve analyses were performed following amplifications. At the end of the cycles, melting temperatures of the PCR products was determined between 70°C and 90°C. The iQ5 software (Bio-Rad) was used for detection of fluorescent signals and melting temperature calculations. Quantification of selected mRNA transcript abundance was performed using the comparative threshold cycle (CT) method. The difference in abundance of mRNA for the genes was determined by analysis of variance.

**Table 1:** Information regarding the specific primers used for the qPCR.

Primer	Sequences (5’→3’)	GenBank accession number	Product Length (bp)
*TYR*	Forward: TGGGGAGTGCAAGTTTG	NM_204160.1	191
	Reverse: TGGAGCCGTTGTTCATCT		
*TYRP1*	Forward: CAGAAGCTCAGTTCCCTCG	NM_205045.1	226
	Reverse: TGGTTGAAGAAGCGTATGG		
*ASIP*	Forward: ATCTCCCACCCATCTCCAT	NM_001115079.1	156
	Reverse: TGAAGTTTGGCACGCAGT		
*KIT*	Forward: CACTCCGCCTTCCACTCAA	NM_204361.1	127
	Reverse: TCTTCTTCCAGATGCCACTCAA		
*MC1R*	Forward: TCCGTCGTGTCCTCCCTCT	NM_00103146.1	262
	Reverse: CCAGCGCGAACATGTGAA		
*OCA2*	Forward: CCAAGCAGGAACTGAGGAGGCA	XM_004938466.1	90
	Reverse: AGGAGACCAGAACAACAAGGCAGAT		
*MITF*	Forward: TGTGACTGAACCAACTGGCACTTAC	NM_205029.1	157
	Reverse: TGCTCCGCCTGCTACTCGTT		
*KITLG*	Forward: AGAGAATGATTCCAGAGTCGCTGTC	NM_001105315.1	97
	Reverse: GCTAGTATTACTGCCAATGCTGTCA		
*β-actin*	Forward: AGGCGAGATGGTGAAAG	NM_205518.1	282
	Reverse: CACGCTCCTGGAAGATAG		

### Statistical Analysis

In this study, the skin from three females was used to prepare one pooled RNA sample for each group of white or black skin. Two cDNA libraries were then constructed to perform the Illumina2000 deep sequencing. The reference genome used in this study can be found at Gallus-gallus.Galgal14.72.dna.toplevel.fa.gz. The significance level was |log2 (Fold change)| >1 with a p value < = 0.05. It ensures an accurate selection of the differentially expressed genes. The relative amount of mRNA expression of each gene (expressed as adjusted Ct value) was analyzed by the Stratagene iQ5 system. Adjusted cycle threshold (C (t)) values were calculated by following equation: CT value = 2^-ΔΔt^. The analysis of variance was performed with SPASS Version 19.0 software. Differences were considered significant at P value < 0.05.

## Results

### Illumina Draft Reads

In this study, we set up two libraries. One was of black skin, another was of white skin. The schematic of the Illumina2000 deep sequencing and analysis from the two libraries are shown in Table 2.

**Table 2:** Summary of the valid reads from the two libraries via Illumina2000 deep sequencing.

Reads	Samples			
	BS		WS	
Total valid reads	59,201,268	100%	55,396,942	100%
Alignment	25,589,116	43.22%	24,285,949	43.84%
Unmapped	33,612,152	56.78%	31,110,993	56.16%

### Differentially Expressed Genes

**Fig 1:**
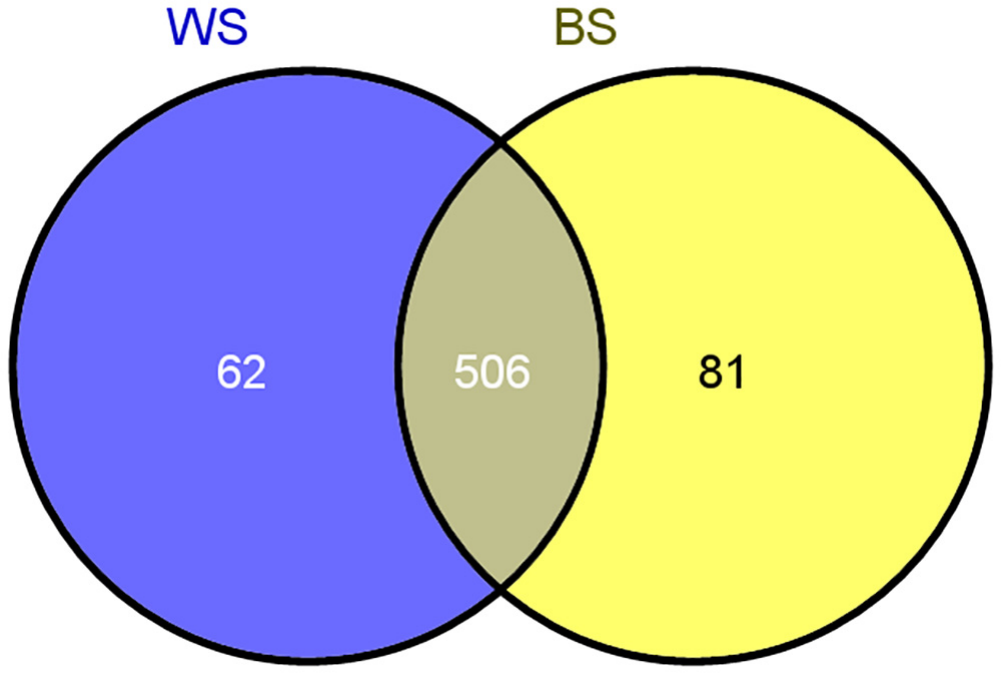
The differentially expressed genes that are unique or shared between WS and BS. WS refers to the white-skinned group. BS refers to the black-skinned group. The numbers in each section of the figure indicate the number of differentially expressed genes in the indicated comparison.

A comparison of the gene expression showed that a total of 649 unigenes were differentially expressed between WS and BS (|log2 (Fold change)| > 1, p value </ = 0.05), with 314 up-regulated genes and 335 down-regulated genes. A total of 162 significantly expressed novel genes were identified between WS and BS (WS vs. BS), consisting of 73 genes that were up-regulated (including 4 highly expressed genes that are expressed exclusively in the black-skinned chickens) and 89 genes that were down-regulated. Moreover, 506 unigenes were significantly differentially expressed in both WS and BS. In addition, 62 and 81 unigenes that were uniquely expressed in WS and BS, respectively. All of the differentially expressed genes are illustrated in Fig 1.

**Fig 2:**
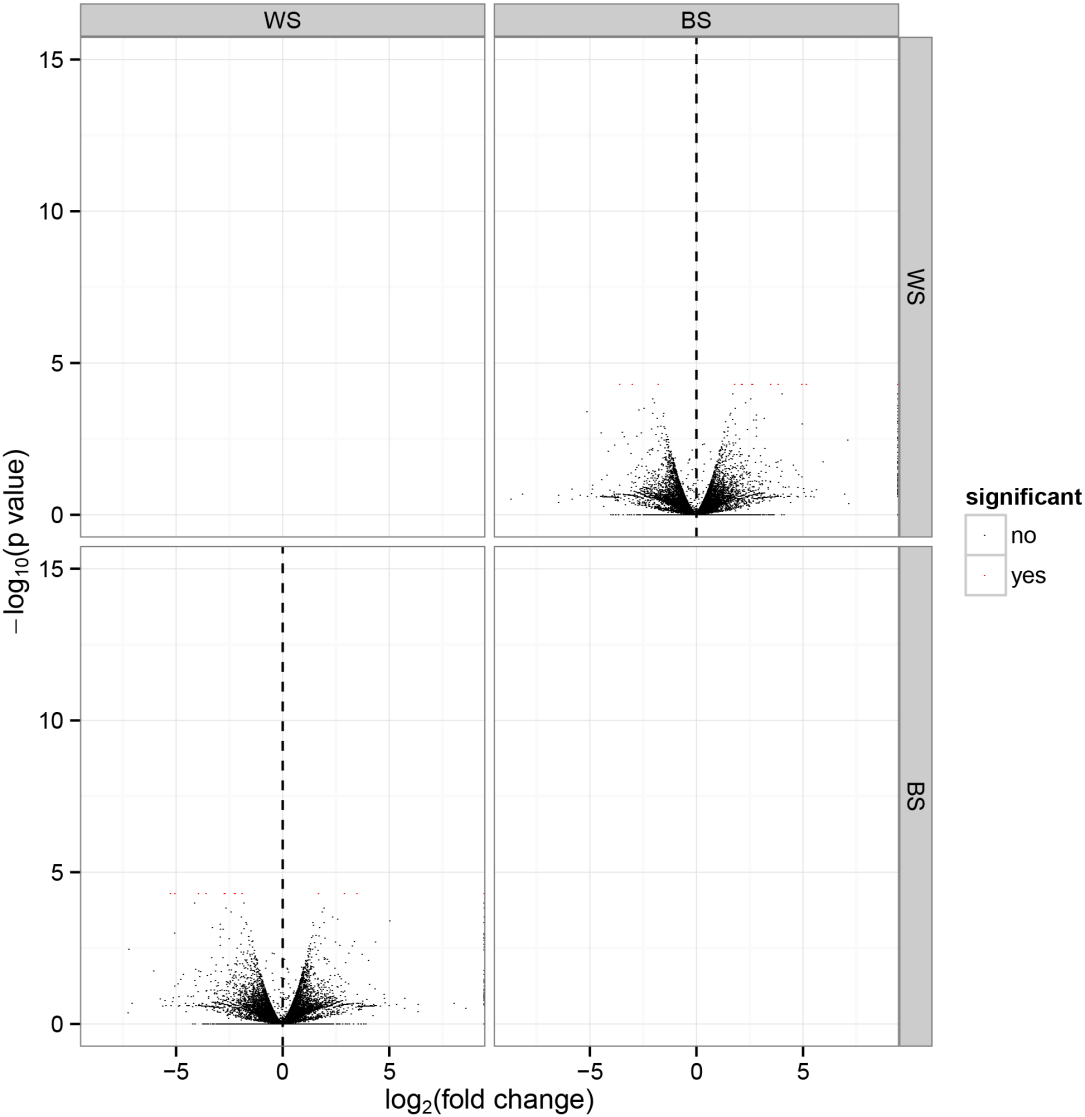
Volcano plots exploring the relationship between the fold change and the level of significance. The red plot represents significance; the black plot represents no significance; log_2_ (Fold Change) is equal to log_2_ (WS value / BS value).

M vs. A plots can be useful to determine any systematic bias that may be present between conditions (A = log2 (Fold change); M = —log10 (P value)). Therefore, we used the log2 (Fold change) value combined with the—log10 (p value) to assess every gene expressed between WS and BS. Volcano plots exploring the relationship between the fold change and the significance are shown in Fig 2.

**Fig 3:**
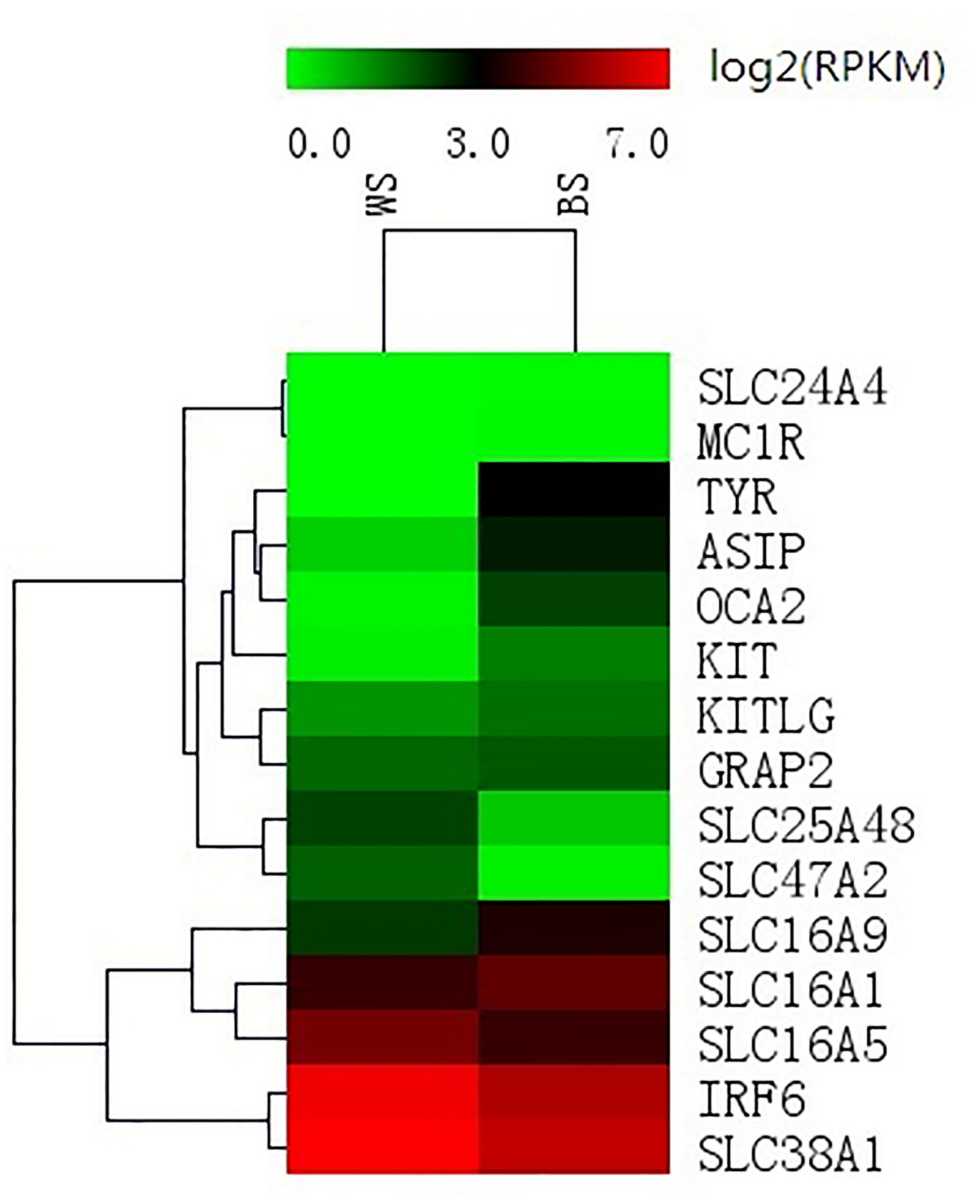
A heat-map exploring the differences in gene expression between WS and BS. Different colors represent different expression levels; a darker color represents higher expression and a greater log2 (RPKM) value.

A heatmap was produced to explore the differences in gene expression. In a heatmap, different colors represent different expression levels, and a darker color represents greater expression. There are many genes with well-known roles in pigmentation that have been identified in previous genome-wide association scans (e.g., *ASIP*, *OCA2*, *TYR*, *MC1R*, *KITLG*, *TYRP1*, *SLC24A4* and *KIT*). Several of these genes showed significant differences in expression between the white- and black-skin samples used in this study; *TYR*, *OCA2*, *ASIP* and *KIT* are shown in Fig 3. *MC1R*, *KITLG*, *GRAP2*, and *IRF6* also showed no differences in expression. From the SLC family, *SLC16A9* and *SLC16A5* were significantly up-regulated (WS vs. BS). However, *SLC16A1*, *SLC25A48* and *SLC47A2* were significantly down-regulated (WS vs. BS), and *SLC24A4* and *SLC38A1* showed no differences in expression between the two skin colors. The expression of *TYRP1* was not detected in either skin color.

### Validation of the Differentially Expressed mRNA in the Chicken Skin

**Fig 4:**
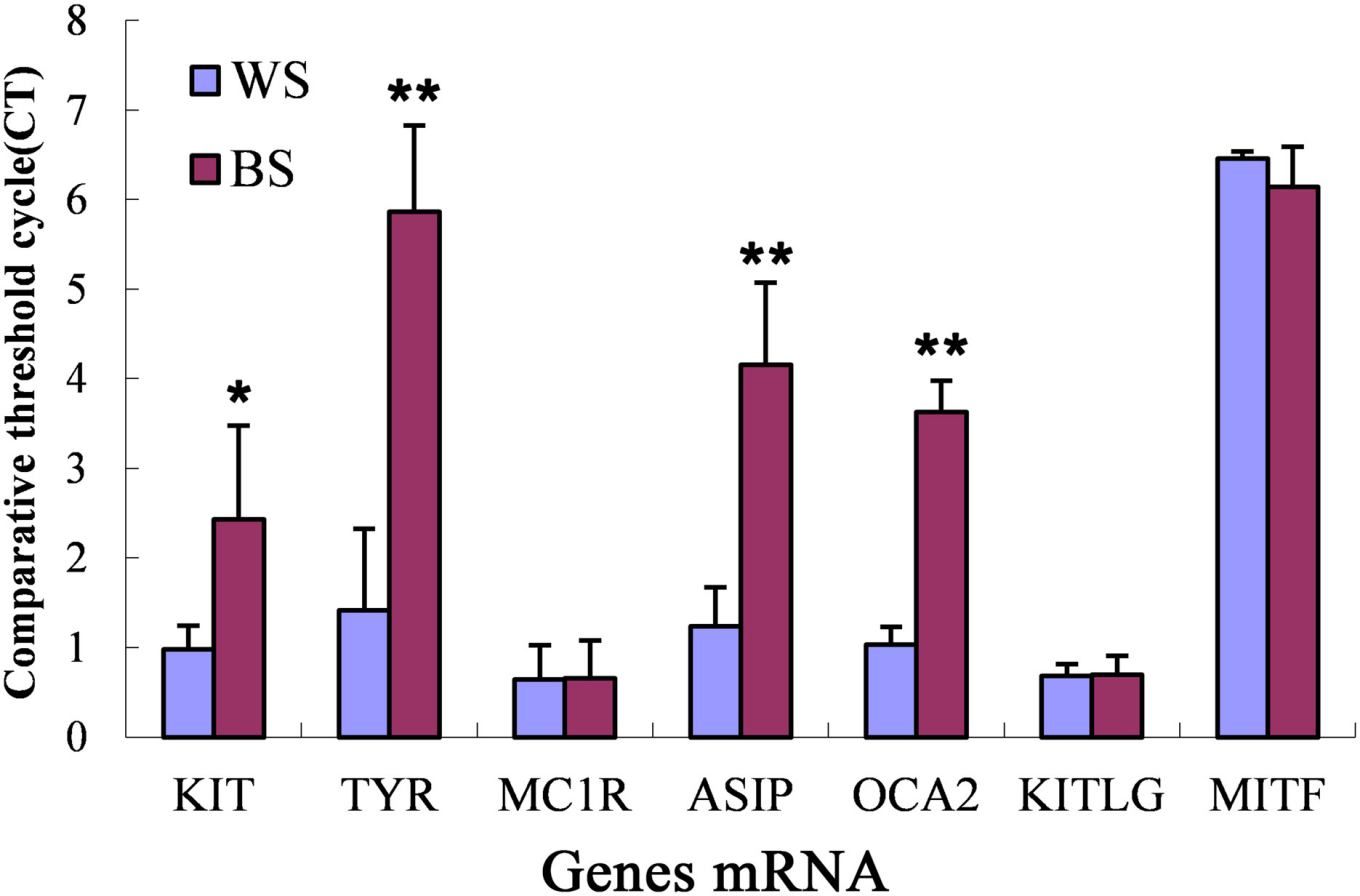
Quantification of the mRNA levels from the RNA extracted from the skin of black (black chicken, red bars) or white chickens (white bars). The bars represent the adjusted ct values; therefore, higher values represent higher mRNA levels. The error bars are the S.E. for 6–8 samples from each skin color group. * 0.01<p<0.05 ** p<0.01.

The expressions of *ASIP*, *TYR*, *KIT*, *TYRP1*, *MC1R*, *OCA2*, *KITLG* and *MITF* were validated using real-time quantitative polymerase chain reaction (qPCR). Each RT-PCR experiment contained triplicates of 10 test individuals (5 birds per color), one no-template-control, and a log10 dilution series. Samples were randomly assigned to PCR plates. The results indicated that there were no significant differences in the expression of *MC1R*, *KITLG* and *MITF* between the black and white skin samples (Fig 4), but the expression of *TYR*, *KIT*, *OCA2* and *ASIP* was significant (P value < = 0.05); the results of the qPCR were consistent with the RNA-seq.

### KEGG Analysis of the Pathways

**Fig 5:**
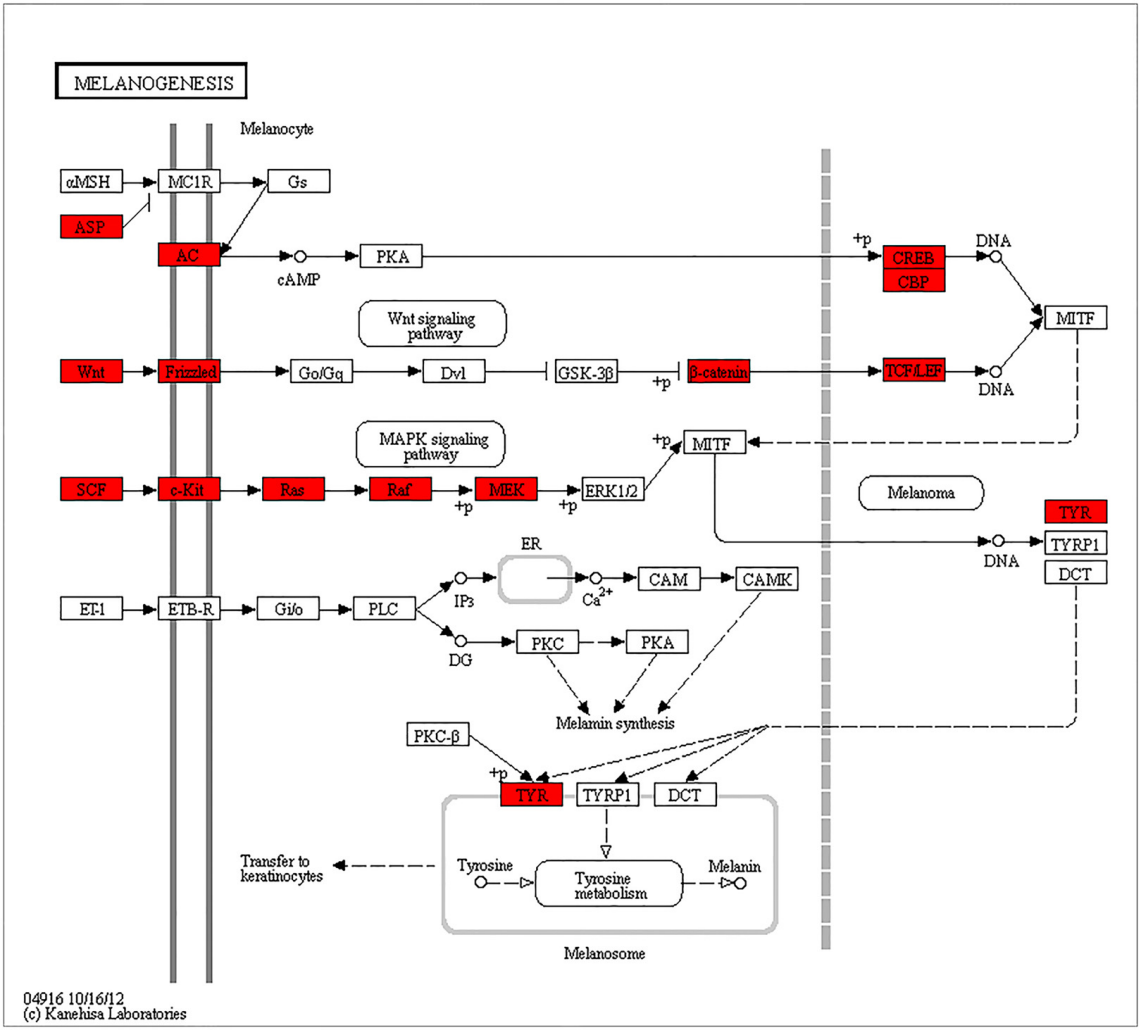
The differentially expressed skin color genes identified in the analyzed chicken skin and their involvement in the melanogenesis pathway. The genes with a red frame are upregulated in the black skin compared to the white skin.

A KEGG enrichment analysis showed that the differentially expressed genes are significantly involved in six predicted pathways (p value </ = 0.01), including steroid hormone biosynthesis, vascular smooth muscle contraction, phenylalanine metabolism, glycolysis / gluconeogenesis, calcium signaling pathway and melanogenesis. All of the pathways and the related information are described in Table 3. In this study, we paid more attention on the pathway of melanogenesis (Fig 5).

**Table 3:** The KEGG enrichment analysis showing that the differentially expressed genes are significantly involved in the predicted pathways.

Pathway names	S gene	TS gene	B gene	TB gene	P value
Steroid hormone biosynthesis	3	649	12	18608	**0.0073**
Vascular smooth muscle contraction	5	649	38	18608	**0.0098**
Phenylalanine metabolism	2	649	8	18608	**0.0296**
Calcium signaling pathway	6	649	71	18608	**0.0373**
Glycolysis / Gluconeogenesis	3	649	23	18608	**0.0445**
Melanogenesis	4	649	40	18608	**0.0498**

Note: S gene represents the number of significant genes that were expressed; TS gene represents the total number of significant genes that were expressed; B gene represents the number of unigenes involved; TB gene represents the total number of unigenes.

A total of 40 differentially expressed genes are involved in the melanogenesis pathway, which plays an important role in animal pigmentation (Table 3). Three crucial genes were identified in this pathway: *KIT*, *TYR*, and *ASIP*, which were significantly up-regulated in BS (p value </ = 0.01). In contrast, expressions of the *MC1R* and *MITF* gene were not significantly different between BS and WS (P value>0.05) (Fig 3).

### GO enrichment Analysis

**Fig 6:**
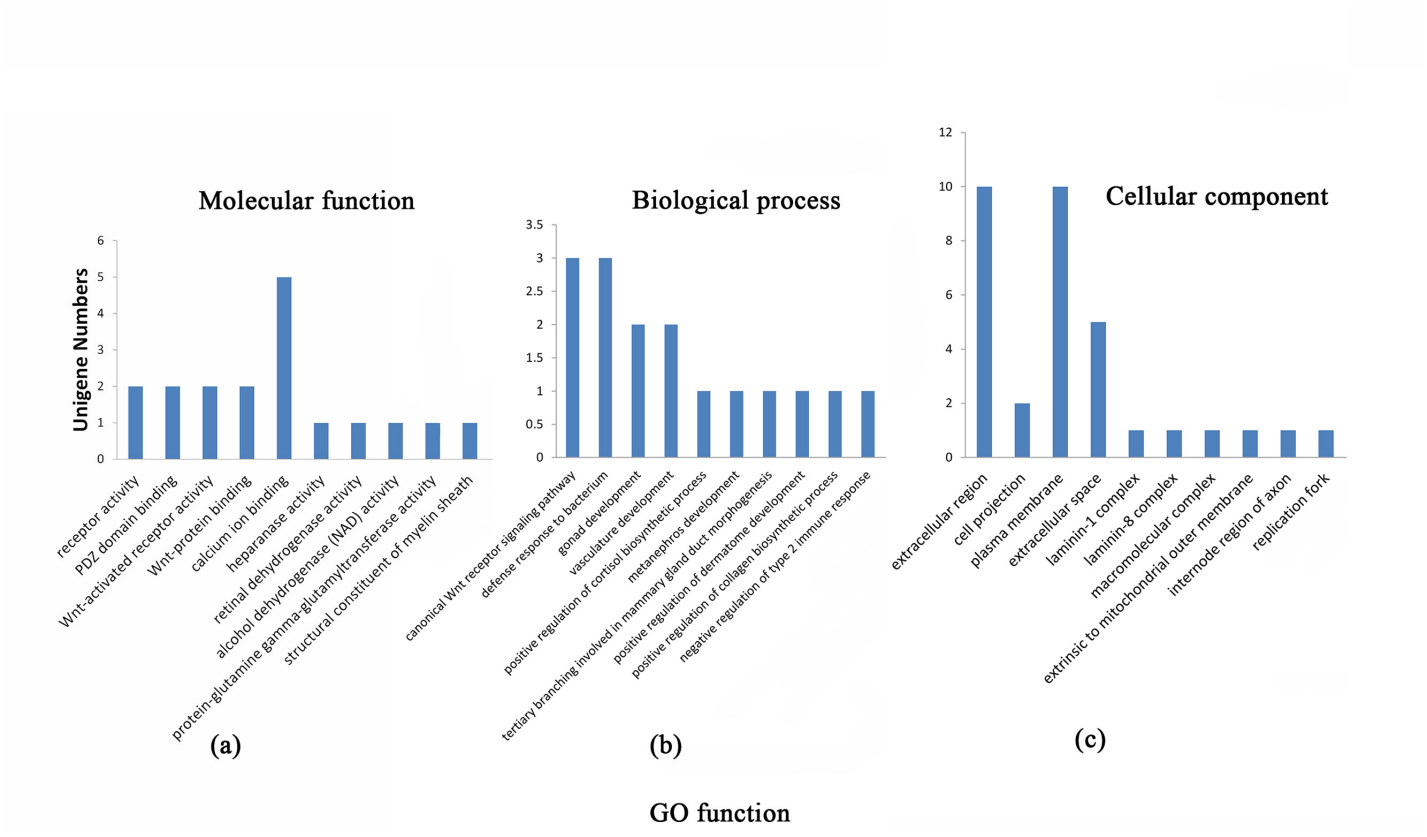
Significantly different GO terms correspond to different categories in the analyzed chicken skin. Note: a. The significantly different GO terms in the molecular function category; b. The significantly different GO terms in the biological process category; c. The significantly different GO terms in the cellular component category.

We analyzed the GO (gene ontology) enrichment of the identified genes. A total of 649 significantly different genes were mapped to the Gene Ontology database (http://www.geneontology.org/), and a total of 222 GO terms were obtained, including 77 significantly different terms (p value < 0.05). These terms were categorized into one of three categories: molecular function, biological process and cellular component (Fig 6). In the molecular function category, 10 significant unigenes are involved in calcium-ion binding (p value = 0.025). The canonical Wnt receptor signaling pathway is significantly different (p value = 0.0042) in the biological process category. In the cellular process category, the extracellular region is the most significant (p value = 0.0001), containing 10 significantly different unigenes.

## Discussion

The mechanisms of melanogenesis are complex, and a number of recent publications have summarized the current understanding of the process of melanin production in the skin [15]. A number of studies, including GWAS, have identified numerous polymorphisms controlling human hair, eye and skin color, such as *MC1R*, *OCA2*, *TYR*, *TYRP1*, *TYRP2*, *ASIP*, *IRF4*, *SLC24A4* and *SLC45A2* [6–8]. A large number of single nucleotide polymorphisms (SNPs) have been identified, very few SNPs have been examined in relation to pigmentary phenotypes. It is well known that the rate-limiting enzyme for the formation of melanin is tyrosinase (*TYR)*, which is responsible for several oxidative steps in the synthesis of melanin [16, 17]. In our study, among the differentially expressed skin color genes, *TYR* showed the greatest level of differential expression in the black versus white chicken skin. This is consistent with studies of sheep coat color [18, 19]. *KIT* is a receptor tyrosine kinase primarily composed of five extracellular immunoglobulin-like domains, a transmembrane domain and two intracellular tyrosine kinase domains. A new study showed that a novel splicing mutation in *KIT* results in piebaldism and auburn hair color in humans [20]. In regards to the important role of *KIT* in UVB-induced melanogenesis in the epidermis [21], more and more studies are focusing on this gene and the potential to lighten human skin color through the inhibition of *KIT* expression. In this study, the expression of the *KIT* gene was also significantly greater in the black-skinned compared to the white-skinned chickens. Black skin color is known to be due to an increase in the ratio of melanin relative to the white skin color. Previous studies revealed that *MITF* (microphthalmia-associated transcription factor) is involved in the development of melanocytes. The present study described the mutation at the *MITF* gene responsible for the plumage color both in the Japanese quail and gallus. The semi dominant B mutation results from a premature stop codon caused by a 2 bp deletion in exon11 of *MITF* [9]. The latest report indicated that there was alternative splicing of *MITF* gene in the skin of sheep [22]. It will provide new way to investigate the different color skin of chickens.

Melanocortin-1 receptor (*MC1R*) is responsible for binding melanocyte stimulating hormone (*MSH*), which is expressed by stressed keratinocytes, and initiating the cascade of melanogenesis [23]. *MC1R* has classically been considered to play a role in and be the irreplaceable target involved in regulating mammalian skin pigmentation and hair color [24–26]. Despite the fact that more than 100 variants of the *MC1R* gene have been identified to date, the consequences of these variants on the physiological function of *MC1R* have been defined only partially [27]. Kinsler et al. (2012) hypothesized that if *MC1R* variants are associated with the growth of moles, the variants might also be associated with fetal growth in general [28]. It has been suggested that *MC1R* is likely to play an important role in the regulation of the immune system. Recent results have also shown that *MC1R* decreases inflammation both in vitro and in vivo and may be part of a therapeutic signaling pathway against inflammatory diseases [29]. All these findings expand on the known variations associated with the *MC1R* gene. More and more studies described that the *MC1R* gene had multiple pleiotropic effects that extend well beyond pigmentation. It can be summarized that *MC1R* does not stop at coloration. Instead, it has been proposed that this gene may also affect behavior, immune function, the nervous system and stress responses [30]. Therefore, many researchers have begun to pay more attention to the role of the *MC1R* gene in pigmentation, and recent studies have demonstrated new findings in regards to *MC1R*. Interestingly, the gene expression of *MC1R*, which has been identified in several studies as a gene related to pigmentation, showed no difference between the white and black skin samples. Three cosmetically important skin lightening agents, hydroquinone (HQ), kojic acid (KA), and niacinamide (NA) make up the bulk of the lightening ingredients in cosmetics, and an examination of these skin lighteners found that all of them led to marked increases in the expression of the *TYR* gene in the melanocytes but not *MC1R* [31]. Another recent study found that there is no association between *MC1R* polymorphisms and plumage coloration in the zebra finch [32], which is consistent with a study that was performed in leaf warblers. Another recently study also indicated that the coat color variation observed in rhesus macaques (variation from light to dark) is unlikely to be due to differences in the expression levels of the *MC1R* gene [33]. In a recent microarray experiment, no significant differences in the expression of *MC1R* were observed between the white skin and black spots of sheep. Instead, the researchers found many new differentially expressed genes that are likely to be involved in the formation of the black spots [34]. Similarly and to our surprise, *MC1R* showed no significant differences in expression between the black-skinned and white-skinned Lueyang chickens used in this study. *MC1R* is constitutively active so that there is no difference in expression between the two morphs. The specific function and role of *MC1R* in pigmentation still requires further research.

In a more recent paper, *TYRP1* was found to show higher expression in the skin of black-coated sheep versus white-coated sheep [19]. However, in our study, the expression of *TYRP1* was not detected in either skin color. The results of our RNA-sequence and the subsequent validation were consistent, but these results still require additional research. The gene for agouti signaling protein (*ASIP*) is centrally involved in the expression of coat-color traits in animals. Previous studies have shown that the *ASIP* gene is responsible for the skin color of both white- and black-coated sheep [18], and a mutation in *ASIP* causes the black-and-tan pigmentation phenotype observed pigs [35]. Consistent with these previous studies, *ASIP* was also found to show significantly different expression levels in the black-skinned chickens versus the white-skinned chickens used in this study. However, in the pathway of melanogenesis (Fig 5), It indicted that *ASIP* (*ASP*) gene was suppressed *MC1R* gene. In epidermal tissues, binding of *ASIP* to *MC1R* leads to the down-regulation of genes such as *MITF* and *TYR*. Finally, the production of pheomelanin would be reduced. In this study, the expression of the *ASIP* gene was also significantly greater in the black-skinned compared to the white-skinned chickens. So, it can suppress the expression of *MC1R* gene in black-skinned chickens. Whether it is the main reason for explaining *MC1R* gene had no significant difference between black- and white-skinned chickens or not, which need more investigations. Oculocutaneous albinism (*OCA*) is the most commonly inherited skin pigmentation disorder. Oculocutaneous albinism type 2 (*OCA2*), the most common *OCA* type, is caused by mutations in the *OCA* gene. The recent discovery of a novel *OCA2* gene variant for melanoma risk in a familial melanoma pedigree provides evidence for the detrimental effects of *OCA2* gene mutations on pigmentation, which supports the existing GWAS data regarding the relevance of the *OCA2* gene in melanoma predisposition [36]. There are exceptions for both *OCA2* and *MC1R*, which also harbor non-synonymous mutations, rs1800414 (His615Arg) in *OCA2* and rs885479 (Arg163Gln) in *MC1R*, which have been associated with skin pigmentation in East Asian populations [37–39]. However, in sheep skin, the genes associated with *OCA* were expressed but the majority did not show any differential expression associated with the coat color [39]. *OCA2* showed significantly different expression between the white- and black-skinned chickens used in this study, and the expression of *OCA2* was up-regulated in black chick, which proving that *OCA2* is closely related to skin color.

The GO and KEGG pathway analysis of the differentially expressed genes revealed that the majority were associated with cell functions and cell components. Of particular interest were the pathways related to pigmentation and melanogenesis. In this study, in terms of the molecular function category, 4 significant genes were found to be involved in melanogenesis, and 10 significant unigenes are involved in calcium-ion binding (p value = 0.025). In the biological process category, the canonical Wnt receptor signaling pathway showed a significant difference as well (p value = 0.0042). Both of these categories are related to pigmentation during the process of melanogenesis. In melanogenesis pathway, Wnt signaling pathway and Ca^2+^ had important roles during melamin synthesis (Fig 5). All these results provide strong evidence that there is a significant difference in the level of melanogenesis between black- and white-skinned Lueyang chickens. However, further investigation is still needed to confirm the regulatory relationships of these genes.

## Conclusion

In summary, this is an original report of the transcriptome analysis of the skin from chickens with white or black skin color. The present study described and revealed a set differentially expressed known and novel genes found in chicken skin that are potentially related to skin color and other physiological functions. The *MC1R* gene showed no difference in expression between chickens with black versus white skin color, and it is of particular interest for future studies to elucidate its functional roles in the regulation of skin color. The results provide a valuable theoretical basis for future breeding schemes for Lueyang chickens and a foundation for research regarding the manipulation of skin color.

## References

[1] Sturm RA, Duffy DL. Human pigmentation genes under environmental selection. Genome biology. 2012;13(9):248 Epub 2012/11/01. 10.1186/gb-2012-13-9-248 23110848 PMC3491390

[2] Dereure O. Drug-induced skin pigmentation. Epidemiology, diagnosis and treatment. American journal of clinical dermatology. 2001;2(4):253–62. Epub 2001/11/14. . 11705252 10.2165/00128071-200102040-00006

[3] Jablonski NG, Chaplin G. Human skin pigmentation as an adaptation to UV radiation. P Natl Acad SciUSA. 2010;107:8962–8. 10.1073/pnas.0914628107 ISI:000277553800010. PMC3024016 20445093

[4] Ducrest AL, Keller L, Roulin A. Pleiotropy in the melanocortin system, coloration and behavioural syndromes. Trends in ecology & evolution. 2008;23(9):502–10. 10.1016/j.tree.2008.06.001 . 18644658

[5] Hart KL, Kimura SL, Mushailov V, Budimlija ZM, Prinz M, Wurmbach E. Improved eye- and skin-color prediction based on 8 SNPs. Croatian Medical Journal. 2013;54(3):248–56. 10.3325/cmj.2013.54.248 WOS:000321410700006. 23771755 PMC3694299

[6] Duffy DL, Zhao ZZ, Sturm RA, Hayward NK, Martin NG, Montgomery GW. Multiple pigmentation gene polymorphisms account for a substantial proportion of risk of cutaneous malignant melanoma. J Invest Dermatol. 2010;130(2):520–8. 10.1038/jid.2009.258 19710684 PMC3672059

[7] Nan H, Kraft P, Hunter DJ, Han J. Genetic variants in pigmentation genes, pigmentary phenotypes, and risk of skin cancer in Caucasians. International journal of cancer Journal international du cancer. 2009;125(4):909–17. 10.1002/ijc.24327 19384953 PMC2700213

[8] Sulem P, Gudbjartsson DF, Stacey SN, Helgason A, Rafnar T, Magnusson KP, et al Genetic determinants of hair, eye and skin pigmentation in Europeans. Nature genetics. 2007;39(12): 1443–52. 10.1038/ng.2007.13 . 17952075

[9] Minvielle F, Bed'hom B, Coville JL, Ito S, Inoue-Murayama M, Gourichon D. The "silver" Japanese quail and the MITF gene: causal mutation, associated traits and homology with the "blue" chicken plumage. Bmc Genetics. 2010;11 Artn 15 10.1186/1471-2156-11-15 WOS:000275834700001. PMC2841575 20184729

[10] Yang GL, Fu DL, Lang X, Wang YT, Cheng SR, Fang SL, et al Mutations in MC1R gene determine black coat color phenotype in Chinese sheep. TheScientificWorldJournal. 2013;2013:675382 10.1155/2013/675382 24082855 PMC3776380

[11] Barrett RD. Bad coat, ripped genes: cryptic selection on coat colour varies with ontogeny in Soay sheep. Molecular ecology. 2012;21(12):2833–5. 10.1111/j.1365-294X.2012.05560.x . 22676073

[12] Gratten J, Beraldi D, Lowder BV, McRae AF, Visscher PM, Pemberton JM, et al Compelling evidence that a single nucleotide substitution in TYRP1 is responsible for coat-colour polymorphism in a free-living population of Soay sheep. Proceedings Biological sciences / The Royal Society. 2007;274(1610):619–26. 10.1098/rspb.2006.3762 17254985 PMC2197217

[13] Li YL, Zhu XP, Yang L, Li JY, Lian ZX, Li N, et al Expression and network analysis of genes related to melanocyte development in the Silky Fowl and White Leghorn embryos. Molecular biology reports.2011;38(2):1433–41. 10.1007/s11033-010-0248-2 WOS:000286472600093. 20848220

[14] Emaresi G, Ducrest AL, Bize P, Richter H, Simon C, Roulin A. Pleiotropy in the melanocortin system: expression levels of this system are associated with melanogenesis and pigmentation in the tawny owl (Strix aluco). Molecular ecology. 2013;22(19):4915–30. 10.1111/mec.12438 . 24033481

[15] Plonka PM, Passeron T, Brenner M, Tobin DJ, Shibahara S, Thomas A, et al What are melanocytes really doing all day long…? Exp Dermatol. 2009;18(12):1096- doi: 10.1111/ j.1600-0625.2009.00985.xISI:000271973700053. 10.1111/j.1600-0625.2009.00912.x PMC2792575 19659579

[16] Parvez S, Kang MK, Chung HS, Cho CW, Hong MC, Shin MK, et al Survey and mechanism of skin depigmenting and lightening agents. Phytotherapy Research. 2006;20(11):921–34. doi: 10.1002 /Ptr.1954 WOS:000242254000001. 16841367 10.1002/ptr.1954

[17] Olivares C, Solano F. New insights into the active site structure and catalytic mechanism of tyrosinase and its related proteins. Pigm Cell Melanoma R. 2009;22(6):750–60. doi: 10.1111/ j.1755-148X.2009.00636.x WOS:000270830600011. 19735457 10.1111/j.1755-148X.2009.00636.x

[18] Norris BJ, Whan VA. A gene duplication affecting expression of the ovine ASIP gene is responsible for white and black sheep. Genome research. 2008;18(8):1282–93. 10.1101/gr.072090.107 WOS:000258116100009. 18493018 PMC2493430

[19] Fan RW, Xie JS, Bai JM, Wang HD, Tian X, Bai R, et al Skin transcriptome profiles associated with coat color in sheep. BMC genomics. 2013;14 Artn 389. 10.1186/1471-2164-14-389 WOS:000320727700001. 23758853 PMC3689618

[20] Yang YJ, Zhao R, He XY, Li LP, Wang KW, Zhao L, et al A Novel Splicing Mutation of KIT Results in Piebaldism and Auburn Hair Color in a Chinese Family. Biomed Res Int. 2013 Artn 689756. 10.1155/2013/689756 WOS:000323507600001. PMC3755434 24000325

[21] Yamada T, Hasegawa S, Inoue Y, Date Y, Yamamoto N, Mizutani H, et al Wnt/beta-Catenin and Kit Signaling Sequentially Regulate Melanocyte Stem Cell Differentiation in UVB-Induced Epidermal Pigmentation. J Invest Dermatol. 2013;133(12):2753–62. 10.1038/Jid.2013.235 WOS:000327015400016. 23702581

[22] Saravanperumal SA, Pediconi D, Renieri C, La Terza A. Alternative splicing of the sheep MITF gene:Novel transcripts detectable in skin. Gene.2014;552(1):165–75. 10.1016/j.gene.2014.09.031 WOS:000343846700021. 25239663

[23] Solano F, Briganti S, Picardo M, Ghanem G. Hypopigmenting agents: an updated review on biological, chemical and clinical aspects. Pigment Cell Research. 2006;19(6):550–71. doi: 10.1111/ j.1600-0749.2006.00334.x WOS:000241739400002. 17083484 10.1111/j.1600-0749.2006.00334.x

[24] Schaffler A, Scholmerich J, Buechler C. The role of 'adipotropins' and the clinical importance of a potential hypothalamic-pituitary-adipose axis. Nat Clin Pract Endocrinol Metab. 2006;2(7): 374–83. 10.1038/ncpendmet0197 . 16932320

[25] Lalueza-Fox C, Rompler H, Caramelli D, Staubert C, Catalano G, Hughes D, et al A melanocortin 1 receptor allele suggests varying pigmentation among Neanderthals. Science. 2007;318(5855): 1453–5. 10.1126/science.1147417 . 17962522

[26] Roberts DW, Newton RA, Beaumont KA, Helen Leonard J, Sturm RA. Quantitative analysis of MC1R gene expression in human skin cell cultures. Pigment Cell Res. 2006;19(1):76–89. 10.1111/j.1600-0749.2005.00286.x . 16420249

[27] Dessinioti C, Antoniou C, Katsambas A, Stratigos AJ. Melanocortin 1 Receptor Variants: Functional Role and Pigmentary Associations. Photochem Photobiol. 2011;87(5):978–87. 10.1111/j.1751-1097.2011.00970.x ISI:000295049700004. 21749400

[28] Kinsler VA, Abu-Amero S, Budd P, Jackson IJ, Ring SM, Northstone K, et al Germline melanocortin-1-receptor genotype is associated with severity of cutaneous phenotype in congenital melanocytic nevi: a role for MC1R in human fetal development. J Invest Dermatol. 2012;132(8):2026–32. 10.1038/jid.2012.95 22572819 PMC3398254

[29] Chen W, Li J, Qu H, Song Z, Yang Z, Huo J, et al The melanocortin 1 receptor (MC1R) inhibits the inflammatory response in Raw 264.7 cells and atopic dermatitis (AD) mouse model. Molecular biology reports. 2013;40(2):1987–96. 10.1007/s11033-012-2256-x . 23090482

[30] Gangoso L, Grande JM, Ducrest AL, Figuerola J, Bortolotti GR, Andres JA, et al MC1R-dependent, melanin-based colour polymorphism is associated with cell-mediated response in the Eleonora's falcon. J Evol Biol. 2011;24(9):2055–63. 10.1111/j.1420-9101.2011.02336.x . 21696477

[31] Gruber JV, Holtz R. Examining the Impact of Skin Lighteners In Vitro. Oxid Med Cell Longev. 2013 Artn 702120. 10.1155/2013/702120 ISI:000318923100001. 23738040 PMC3655678

[32] Hoffman JI, Krause ET, Lehmann K, Kruger O. MC1R Genotype and Plumage Colouration in the Zebra Finch (Taeniopygia guttata): Population Structure Generates Artefactual Associations. Plos One. 2014;9(1). ARTN e86519 10.1371/journal.pone.0086519 ISI:000 330570000044. PMC3906038 24489736

[33] Bradley BJ, Gerald MS, Widdig A, Mundy NI. Coat Color Variation and Pigmentation Gene Expression in Rhesus Macaques (Macaca mulatta). Journal of Mammalian Evolution. 2013;20(3): 263–70. 10.1007/s10914-012-9212-3 WOS:000322187200008.

[34] Penagaricano F, Zorrilla P, Naya H, Robello C, Urioste JI. Gene expression analysis identifies new candidate genes associated with the development of black skin spots in Corriedale sheep. Journal of Applied Genetics. 2012;53(1):99–106. 10.1007/s13353-011-0066-9 WOS:000299525700011. 21952730

[35] Drogemuller C, Giese A, Martins-Wess F, Wiedemann S, Andersson L, Brenig B, et al The mutation causing the black-and-tan pigmentation phenotype of Mangalitza pigs maps to the porcine ASIP locus but does not affect its coding sequence. Mammalian Genome. 2006;17(1): 58–66. 10.1007/s00335-005-0104-1 WOS:000234825800007. 16416091

[36] Hawkes JE, Cassidy PB, Manga P, Boissy RE, Goldgar D, Cannon-Albright L, et al Report of a novel OCA2 gene mutation and an investigation of OCA2 variants on melanoma risk in a familial melanoma pedigree. J Dermatol Sci. 2013;69(1):30–7. 10.1016/j.jdermsci.2012.09.016 WOS:000313611600003. 23103111 PMC4775076

[37] Yamaguchi K, Watanabe C, Kawaguchi A, Sato T, Naka I, Shindo M, et al Association of melanocortin 1 receptor gene (MC1R) polymorphisms with skin reflectance and freckles in Japanese. Journal of human genetics. 2012;57(11):700–8. 10.1038/Jhg.2012.96 WOS:000311489500002. 22854540

[38] Abe Y, Tamiya G, Nakamura T, Hozumi Y, Suzuki T. Association of melanogenesis genes with skin color variation among Japanese females. J Dermatol Sci. 2013;69(2):167–72. doi: 10.1016/j. jdermsci.2012.10.016 WOS:000315538800010. 23165166 10.1016/j.jdermsci.2012.10.016

[39] Edwards M, Bigham A, Tan JZ, Li SL, Gozdzik A, Ross K, et al Association of the OCA2 Polymorphism His615Arg with Melanin Content in East Asian Populations: Further Evidence of Convergent Evolution of Skin Pigmentation. PLoS genetics. 2010;6(3). ARTN e1000867 10.1371/journal.pgen.1000867 WOS:000276311400021. 20221248 PMC2832666

